# Supplementary material for: Temporal responses of bumblebee gustatory neurons to sugars
Source: iScience. 2022 Jun 2;25(7):104499. doi: 10.1016/j.isci.2022.104499 (PMC9207677; doi:10.1016/j.isci.2022.104499)
Supplement: Document S1. Figures S1–S4 and Tables S1 and S2 [file mmc1.pdf]

iScience, Volume *25*

Supplemental information

Temporal responses of bumblebee gustatory neurons to sugars

Rachel H. Parkinson, Sébastien C. Kessler, Jennifer Scott, Alexander Simpson, Jennifer Bu, Mushtaq Al-Esawy, Adam Mahdi, Ashwin Miriyala, and Geraldine A. Wright

Supplemental Figures

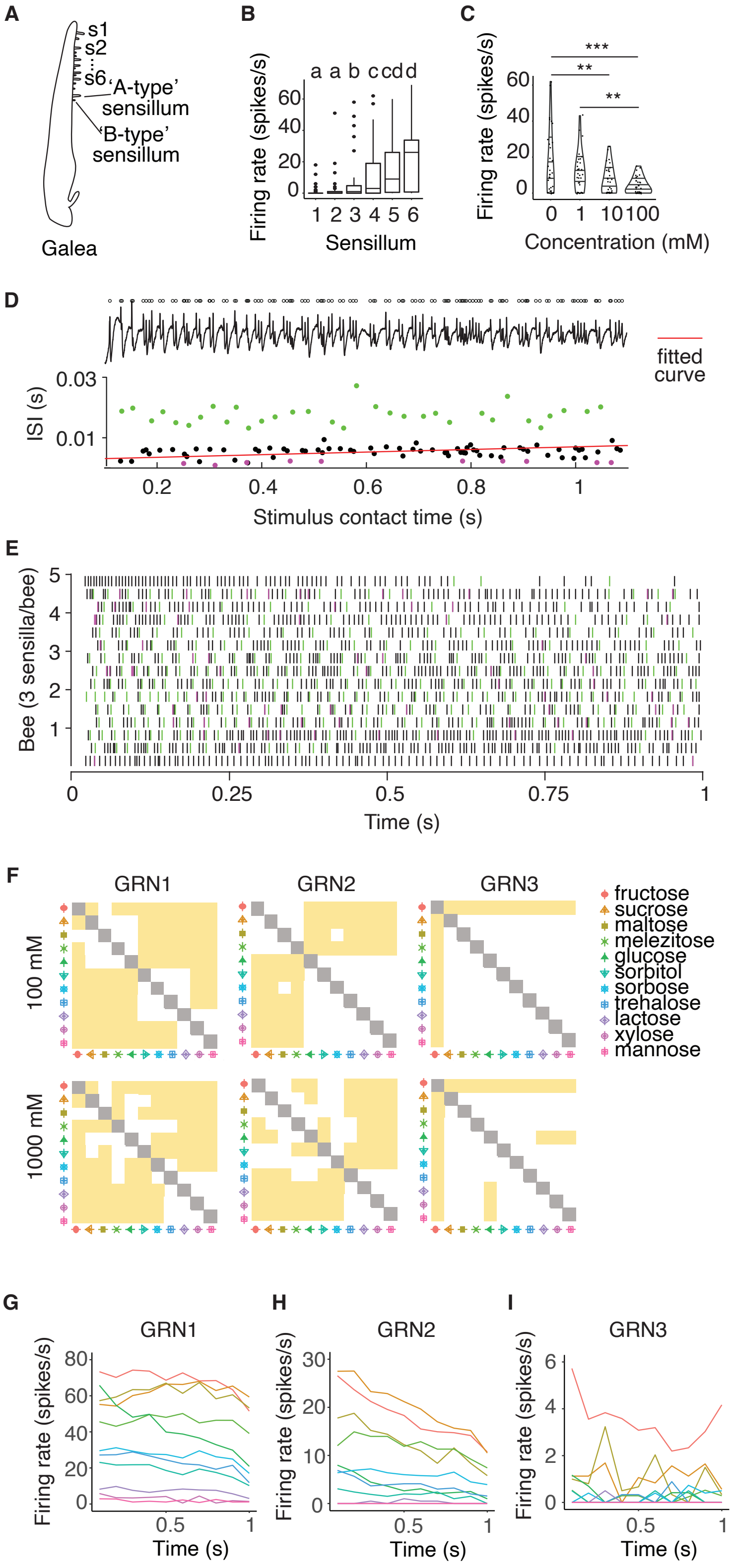

Supplemental Figure 1. Responses of bumblebee galeal GRNs to water and sugars. Related to Figure 1A-J. **A**, Diagram of a bumblebee galea displaying 'A type' and 'B type' sensilla. A-type sensilla are labeled from s1 to s6 from the most distal sensillum. **B**, Average firing rates of the 6 most distal galeal A-type sensilla to water varied by sensillum position (Kruskal-Wallis, χ^2_5 =65.94, p<0.0001). Letters denote pairwise comparisons using Wilcoxon rank sum with continuity correction. **C**, Average firing rate of GRN1 in sensilla 4-6 decreased with increasing concentration for sugars that displayed low valence for GRNs (lactose, xylose and mannose, aligned rank transform ANOVA, $F_{3,217}$ =11.4, p<0.0001). Violin plots show median, 1st and 3rd quartile lines, and individual data points. Asterixis denote post hoc multiple comparisons. **D**, The ISIs versus time were fit to a logarithmic model (red line) to differentiate GRN1 (black points) and GRN2 spikes (green points). GRN3 spikes (magenta) were identified by their <2.5 ms ISIs (and not preceding a 10-20 ms ISI, i.e. a GRN2 spike). **E**: Raster plots of GRN1 (black), GRN2 (green) and GRN3 (magenta) spikes over 1 s stimulation with 100 mM fructose. Data represents single sensillum responses (sensilla 1-3) from 5 bees. **F**, Results of post hoc multiple comparisons (estimated marginal means) between sugars at 100 mM (top row) and 1000 mM (bottom row) for GRNs 1-3. Yellow squares represent significant differences between sugars with a 0.95 confidence level, and the Sidak correction for multiple comparisons. **G-I**, Firing rate histograms over 1 second of stimulation with 100 mM concentrations of sugars for GRN1 (B), GRN2 (C), and GRN3 (D). Mean firing rates were calculated for spikes in 100 ms bins. Note the changing y-axis limits between panels.

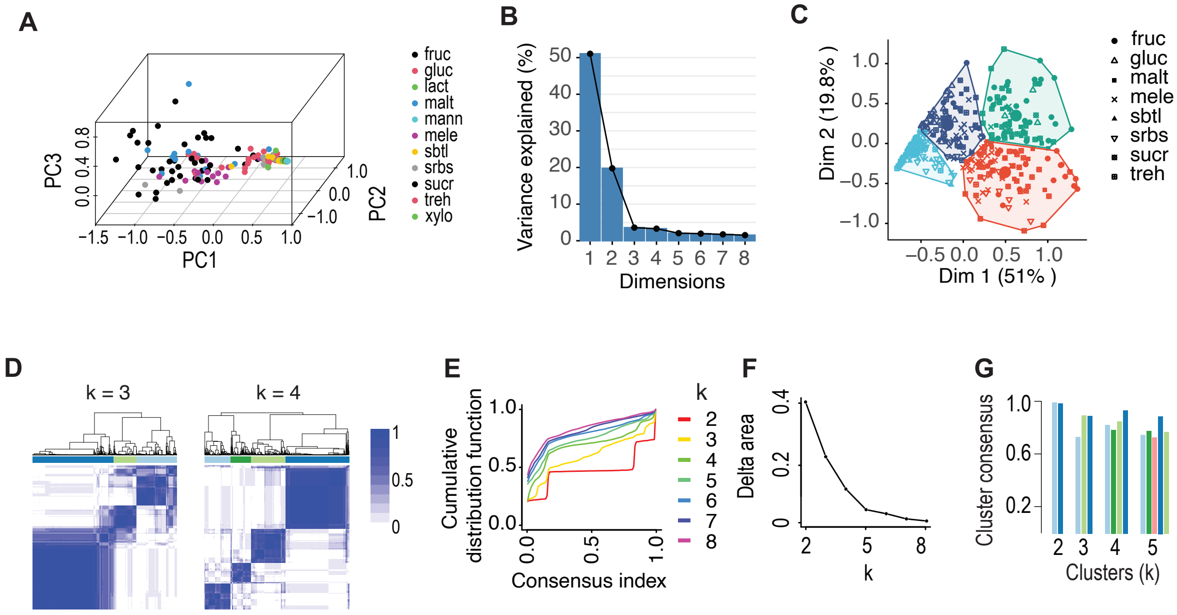

Supplemental Figure 2. Clustering of GRN responses to sugars reveals 4 groupings. Related to Figure 1K and STAR Methods section "Consensus clustering.". **A**, Visualization of the first three principal components (PCs) from a principal component analysis of the binned spike times (100 ms bins) of GRNs 1-3 to sugars at 100 mM. **B**, Scree plot showing the percent of explained variances by PCs 1-8 using binned spike time data for responses of GRNs 1-3 to sugars at 10 − 1000 mM concentrations. **C**, Kmeans clustering of the first 3 principal components (PCs) for all sugars and concentrations from 10 − 1000 mM. **D**, Consensus matrices constructed from iterative consensus clustering algorithms using k-means clustering of the 8 PCs derived from the time series GRN responses. The matrices quantify how consistently certain data points were clustered together over clustering iterations, and the consensus matrix itself is then used to cluster unique GRN responses together and incorporate the information across clustering runs. Matrices show that k=4 clusters results in increased cluster consensus across runs. Color bar indicates matrix consensus from 0 (no consensus) to 1 (consensus on every clustering iteration). **E-G**, Consensus clustering validation: E: Cumulative distribution function (CDF) for the consensus matrix values with k varied from 2 to 8. The CDF displays only modest increases with increasing cluster count after 4 clusters. F: Delta area plot shows the relative change in area under the curve from the CDF plot at k versus k-1, showing there is no appreciable increase in consensus with k¿4. G: Cluster consensus with clusters varying from k=2 to 5 showing the mean pairwise consensus values between a cluster's members, with high values indicating a cluster with high stability. Using these metrics we determined that the greatest cluster consensus is obtained with k=4 clusters.

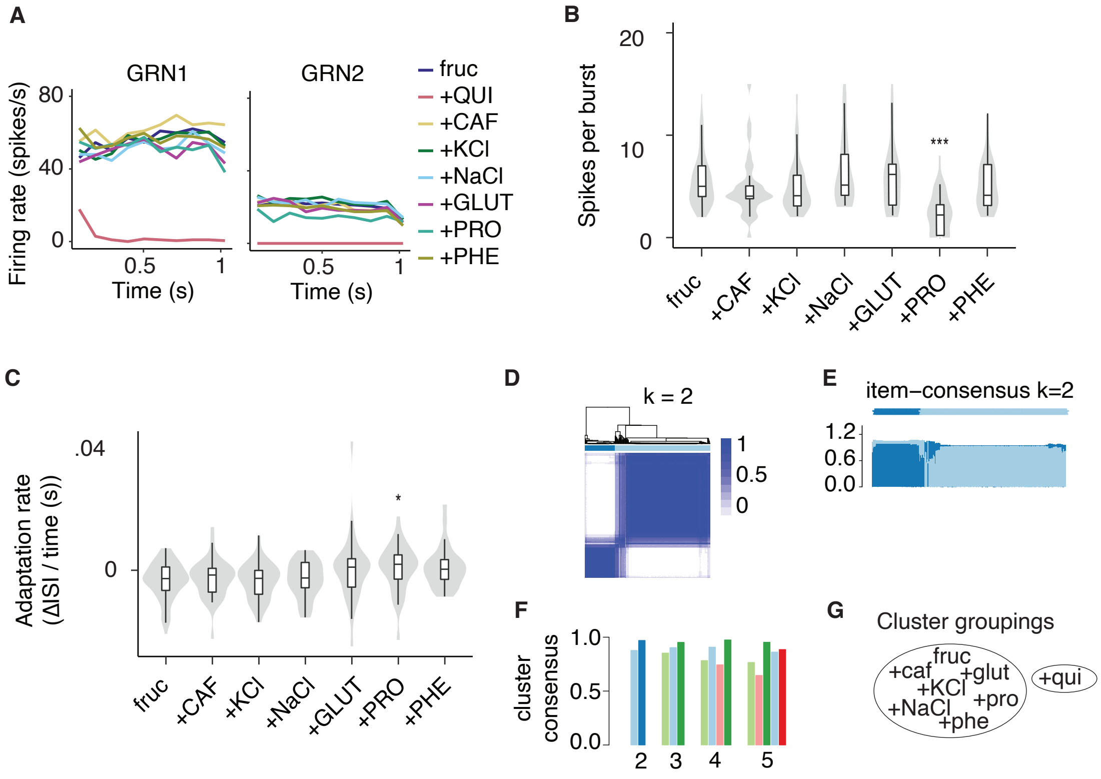

Supplemental Figure 3. GRN responses to fructose mixtures. Related to Figure 3. **A**, Firing rate histograms (spikes in 100ms bins) over 1 s of stimulation for GRN1 and GRN2. Lines represent mean measurements across all sensilla for a given stimulus. **B-C**: Proline affected burst structure and GRN1 adaptation: the maximum number of GRN1 spikes per burst was reduced (B, aligned rank transform ANOVA, $F_{6,261}$ =9.61, $p<0.0001$) and the adaptation rate for GRN1 was increased (C, $F_{7,262}$ =3.61, $p<0.05$). Violin plots show density of response with overlaid boxplots displaying 1st, 2nd and 3rd quartiles with 95% confidence interval whiskers. Asterisk denotes significant effects versus fructose alone. **D-G**, Consensus clustering of GRN responses to fructose mixtures. **D**, Consensus matrix, **E**, item consensus, and **F**, cluster consensus show high levels of consensus with k=2 clusters. **G**, Clustering analysis resulted in the separation of responses in two clusters, with quinine clustering separately from all other responses.

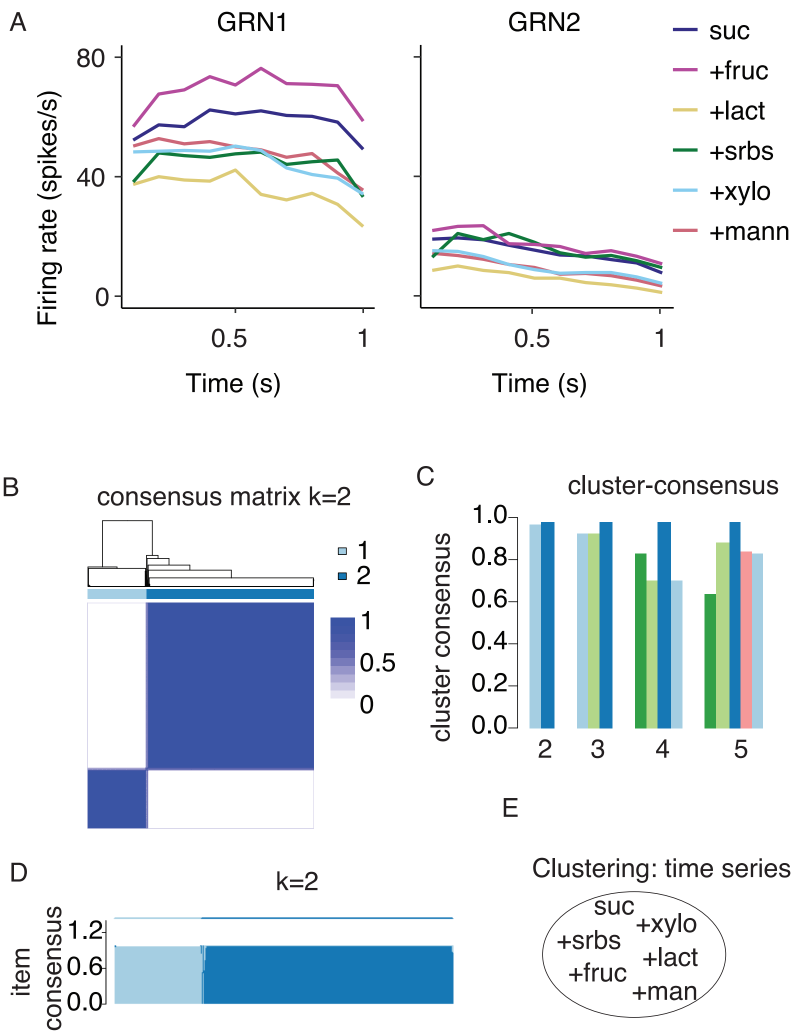

Supplemental Figure 4. Consensus clustering of GRN responses to sucrose mixtures. Related to Figure 4. **A**, Firing rate histograms (spikes in 100ms bins) over 1 s of stimulation for GRN1 and GRN2 in response to sucrose and sucrose mixtures. Lines represent mean measurements across all sensilla for a given stimulus. **B-E**, Consensus clustering of time-series GRN responses with 500 mM sucrose or 500 mM equimolar sucrose mixtures results in k=2 clusters. Consensus matrix (**B**), cluster consensus (**C**) and item consensus (**D**) show high levels of consensus with k=2 clusters. **E**, Responses that clustered together in the two groups were equally distributed amongst stimuli (i.e. clustering represented variation in sensilla responses to a single stimulus) and the responses in the second cluster represented a small proportion of the responses to any stimulus (average of 0.24 across stimuli). Thus, the greatest proportion (0.76) of sensilla responses clustered together in a single group.

Supplemental Tables.

Table 1. Adaptation of GRN1 vs GRN2 over 1 s. Values are the slopes of fitted models corresponding to Figure 1H-J.

Sugar	10 mM	100 mM	1000 mM
fructose	2.264	0.995	0.750
sucrose	2.010	-0.453	0.118
maltose	0.936	0.056	0.398
melezitose	1.683	0.700	0.768
glucose	3.384	5.706	4.747
sorbitol	2.095	3.776	1.613
sorbose	2.095	3.234	1.211

Table 2. PCA factor loadings Loadings shown for PC1 to PC8 for each 0.1 s bin of GRNs 1-3 between 0.1 s and 1 s. Related to STAR Methods section "Consensus clustering."

GRN	Time (s)	PC1	PC2	PC3	PC4	PC5	PC6	PC7	PC8
GRN1	0.1	0.1268	0.3356	-0.2356	0.2763	-0.0042	0.4793	-0.1599	0.1950
	0.2	0.1652	0.3174	-0.1029	0.1741	0.0107	0.2339	0.0340	0.3205
	0.3	0.1859	0.2844	-0.0320	-0.0018	-0.0666	0.1166	0.1059	-0.0317
	0.4	0.2081	0.2578	-0.0770	0.0828	-0.0266	-0.0402	-0.3113	-0.0664
	0.5	0.2025	0.2543	-0.0860	-0.0622	-0.0162	-0.0388	0.1357	-0.2124
	0.6	0.2194	0.2253	-0.0132	-0.0705	0.0401	-0.0596	0.1775	-0.1014
	0.7	0.2140	0.2056	0.0272	-0.1102	0.1374	-0.3526	0.1094	0.2359
	0.8	0.2129	0.1700	-0.0336	-0.0311	-0.2842	-0.1729	-0.0332	-0.2183
	0.9	0.2166	0.1712	-0.0064	-0.1439	0.0004	-0.3293	-0.0509	-0.0703
	1	0.2059	0.1128	-0.0565	-0.0789	0.0395	-0.2501	0.2080	-0.1283
GRN2	0.1	0.3246	-0.0778	0.3700	-0.5031	-0.0071	0.3068	-0.1902	0.1661
	0.2	0.3264	-0.1897	0.1178	-0.2159	0.0739	0.2940	-0.1362	-0.3011
	0.3	0.2875	-0.2078	-0.0105	0.0659	0.2145	-0.0826	-0.2942	0.0076
	0.4	0.2483	-0.2209	-0.1047	-0.1083	0.0155	0.1058	0.4999	0.1439
	0.5	0.2454	-0.2376	-0.0458	0.0984	-0.0056	-0.1168	-0.1478	0.3046
	0.6	0.2196	-0.2451	-0.1558	0.1521	-0.1245	-0.1270	-0.2217	0.0808
	0.7	0.2008	-0.2240	-0.1549	0.2194	-0.2021	0.1633	0.0970	-0.4310
	0.8	0.1893	-0.2045	-0.1600	0.1052	0.3272	-0.0808	0.1975	0.2259
	0.9	0.1685	-0.2005	-0.1705	0.2394	-0.1198	0.0408	0.2460	-0.0001
	1	0.1299	-0.1409	-0.0612	0.1719	-0.1160	-0.0824	-0.1274	0.0527
GRN3	0.1	0.0756	0.0719	0.3748	0.4245	0.6168	0.0089	-0.0263	-0.2976
	0.2	0.0504	0.0268	0.2325	0.2123	-0.1404	-0.0904	-0.0353	0.1117
	0.3	0.0629	0.0093	0.4800	0.1725	-0.2205	0.1105	0.2890	0.1704
	0.4	0.0409	0.0136	0.1894	0.0963	-0.1659	-0.1734	-0.1917	0.1951
	0.5	0.0367	0.0082	0.2336	0.0329	-0.0822	0.0508	0.1543	-0.0329
	0.6	0.0238	0.0200	0.1235	0.1010	-0.0368	-0.2037	-0.0319	0.0251
	0.7	0.0369	0.0132	0.1842	0.1226	0.0760	0.0169	0.1020	0.0305
	0.8	0.0400	-0.0152	0.0893	0.1511	-0.2989	-0.0270	-0.0912	-0.1571
	0.9	0.0462	-0.0313	0.1476	0.0704	-0.1069	-0.0407	0.0188	-0.0168
	1	0.0483	-0.0064	0.2223	0.1566	-0.2402	-0.0522	0.0534	0.0107
